# T Cell Epitope Regions of the *P. falciparum* MSP1-33 Critically Influence Immune Responses and *In Vitro* Efficacy of MSP1-42 Vaccines

**DOI:** 10.1371/journal.pone.0024782

**Published:** 2011-09-13

**Authors:** Kae M. Pusic, Caryn N. Hashimoto, Axel Lehrer, Charmaine Aniya, David E. Clements, George S. Hui

**Affiliations:** 1 Department of Tropical Medicine, Medical Microbiology, and Pharmacology, John A. Burns School of Medicine, University of Hawaii, Honolulu, Hawaii, United States of America; 2 Hawaii Biotech Inc., Aiea, Hawaii, United States of America; Queensland Institute of Medical Research, Australia

## Abstract

The C-terminal 42 kDa fragments of the *P. falciparum* Merozoite Surface Protein 1, MSP1-42 is a leading malaria vaccine candidate. MSP1-33, the N-terminal processed fragment of MSP1-42, is rich in T cell epitopes and it is hypothesized that they enhance antibody response toward MSP1-19. Here, we gave *in vivo* evidence that T cell epitope regions of MSP1-33 provide functional help in inducing anti-MSP1-19 antibodies. Eleven truncated MSP1-33 segments were expressed in tandem with MSP1-19, and immunogenicity was evaluated in Swiss Webster mice and New Zealand White rabbits. Analyses of anti-MSP1-19 antibody responses revealed striking differences in these segments' helper function despite that they all possess T cell epitopes. Only a few fragments induced a generalized response (100%) in outbred mice. These were comparable to or surpassed the responses observed with the full length MSP1-42. In rabbits, only a subset of truncated antigens induced potent parasite growth inhibitory antibodies. Notably, two constructs were more efficacious than MSP1-42, with one containing only conserved T cell epitopes. Moreover, another T cell epitope region induced high titers of non-inhibitory antibodies and they interfered with the inhibitory activities of anti-MSP1-42 antibodies. In mice, this region also induced a skewed TH2 cellular response. This is the first demonstration that T cell epitope regions of MSP1-33 positively or negatively influenced antibody responses. Differential recognition of these regions by humans may play critical roles in vaccine induced and/or natural immunity to MSP1-42. This study provides the rational basis to re-engineer more efficacious MSP1-42 vaccines by selective inclusion and exclusion of MSP1-33 specific T cell epitopes.

## Introduction

The C-terminal fragments of the Merozoite Surface Protein 1 (MSP1) of *P. falciparum*, MSP1-42, is one of the leading candidates for a blood-stage malaria vaccine [1]. MSP1 is a 195kDa protein that is proteolytically processed during schizogony into four smaller fragments: 83 kDa, 30 kDa, 38 kDa, and 42 kDa [2], [3]. The C-terminal 42 kDa protein is then further processed during merozoite invasion into a 33 kDa and a 19 kDa fragment [3]. The 19 kDa fragment (MSP1-19) is carried into the infected erythrocyte by the merozoites, while the 33 kDa fragment (MSP1-33) is released into the blood plasma [4]. Protective immunity induced by MSP1-42/MSP1-19 has been shown to be antibody mediated [5], [6], [7], [8], [9]. Passive transfers of anti-MSP1-42 or MSP1-19 monoclonal and polyclonal antibodies have protected against malaria [10], [11], [12], [13], [14], [15]; MSP1-42 or MSP1-19 specific antibodies may act by inhibiting merozoite invasion [9]. On the other hand, blocking antibodies specific for MSP1-42/MSP1-19 have also been detected, and these antibodies interfere with the activities of parasite inhibitory anti-MSP1-19 antibodies [16]. Vaccination studies with MSP1-42 or MSP1-19 have demonstrated strong or complete protection against blood infections in rodent and monkey models [17], [18], [19], [20], [21]. Monkeys protected by MSP1-42 vaccinations produce parasite inhibitory antibodies [17], [19], [20], thus suggesting that vaccine-induced immunity is also antibody mediated.

Although the above studies have convincingly demonstrated the vaccine potential of MSP1-42/MSP1-19, a Phase II clinical trial using MSP1-42 resulted in no *in vivo* protection [22]. The inability of the MSP1-42 vaccine formulation to induce protection in this clinical trial could be attributed to very low levels (titers) of parasite inhibitory antibodies [22], [23]. Two Phase I trials of MSP1-42 using Alum and Alum+CPG adjuvants also resulted in low levels of inhibitory antibodies [24], [25]. The failure to elicit protective immunity and/or high levels of parasite inhibitory antibodies in these clinical trials may be attributed to a number of factors: a) serum samples from vaccinated individuals have no parasite inhibitory effects suggesting that the MSP1-42 vaccine induced antibodies of the wrong specificity [22], [24]: b) the magnitude of antibody titers induced by the MSP1-42 vaccines were not high enough to have biological activities [23], [24], [26]: c) antibodies were relatively short-lived to confer protection [22], [25]: and d) inadequate induction of memory responses [27]. A better understanding of the vaccine-induced immune response to MSP1-42 may help to overcome these shortcomings and may help to design a more efficacious MSP1-42 vaccine.

Unlike MSP1-42/MSP1-19, there have been few studies on MSP1-33. Studies on MSP1-33 primarily focus on mining T cell epitopes [28], [29], [30] since it has been shown that MSP1-19 does not possess adequate T helper epitopes to stimulate antibody response in a diverse genetic population [29], [31]. Thus, it has been suggested that these T cell epitopes on MSP1-33 may provide cognate helper function specific for anti-MSP1-19 antibody response [29], [30], [31], [32], [33], [34]. It is assumed that MSP1-33 specific T cell epitopes will all contribute positively to the induction of biologically active anti-MSP1-19 antibodies. However, it has been well established in other model systems that T cell epitopes can influence the development antibody response to B cell epitopes [35], [36], [37], [38]. Indeed, previous studies have observed differences in antibody specificity induced by MSP1-19 versus MSP1-42 (ie. MSP1-33 + MSP1-19) [39]. In a genetically diverse population, MSP1-42 is more effective in inducing parasite growth inhibitory antibody responses than MSP1-19 [39]. In addition, in vivo protection induced by MSP1-19 is also regulated by the host's immune response, (IR) genes [31], [33]. Moreover, MSP1-42 induce antibodies that are more broadly cross-reactive with other allelic forms of MSP1-19 than the MSP1-19 fragment [39], suggesting that MSP1-42 may elicit antibodies to additional epitopes [39]. It is possible that MSP1-33, which harbors abundant T cell epitopes, may influence antibody responses induced by MSP1-42. To address this hypothesis, we investigated the ability of T cell epitopes of MSP1-33 to provide help, and whether they can critically influence antibody specificity. Outbred Swiss Webster mice were used to examine the efficacy of eleven recombinant MSP1-42 proteins consisting of truncated segments of MSP1-33 linked to MSP1-19. Additionally, the recombinant subunit proteins, formulated with ISA51, were evaluated in New Zealand White (NZW) rabbits for the induction of parasite growth inhibitory antibodies. Results showed that T cell epitopes of MSP1-33 have a profound influence on MSP1-42 vaccine efficacy.

## Materials and Methods

### Ethics Statement

All experiments involving animals (mice and rabbits) were approved by the University of Hawaii Institutional Animal Care and Use Committee (IACUC). Procedures were designed to inflict minimum pain and distress as possible. The use of animals in experimentation was strictly adhered to the "Guide for the Care and Use of Laboratory Animals" published by the Institute for Laboratory Animal Research (ILAR). Immunized animals were monitored for unusual pain and distress and they would have been euthanized if such symptoms appeared. Euthanasia was performed according to the methods recommended by the American Veterinary Medical Association (AVMA). University of Hawaii's Animal Care Assurance number is A3423-01. For all animal studies, the IACUC approved specific protocol number is 08-389.

### Mouse and Rabbit strains

Outbred Swiss Webster mice (female, 6–8 weeks old) were obtained from four different vendors, Taconic (Albany, NY), Simonsen (San Clara, CA), Harlan Spraque Dawley Inc. (Indianapolis, IA), and Charles River Laboratory (Wilmington, MA) to ensure genetic heterogeneity. New Zealand White (NZW) rabbits (female, 8–10 lbs) were obtained from Western Oregon Rabbit Company (Philomath, Oregon). The use of mice and rabbits were approved by the University of Hawaii's Institutional Animal Care and Use Committee.

### MSP1-specific antibody assays

Mouse and rabbit sera were assayed for anti-MSP1 antibodies (MSP1-42 and MSP1-19) by direct binding ELISAs as previously described [40]. Recombinant MSP1-42 and MSP1-42 antigens used for coating ELISA plates were produced based on *P. falciparum* FUP strain and were obtained from previous studies. The MSP1-42 was expressed in baculovirus [33],and, MSP1-19 was expressed in yeast [32]. Recombinant MSP1-33 was expressed in E.coli and was based on the 3D7 strain, which has identical MSP1-33 sequence as FUP [41]. Briefly, 96-well ELISA plates (Costar, Acton, MA) were coated with the appropriate test antigen at a concentration of 0.4 µg/mL. Plates were then blocked with 1% Bovine Serum Albumin (BSA) in Borate Buffered Saline (BBS). Test sera were serially diluted in 1% BSA/0.5% yeast extract/BBS and then incubated for 60 minutes in the antigen-coated ELISA wells. Wells were washed seven times with High Salt Borate Buffered Saline (HSBBS) and incubated for 60 minutes with horseradish peroxidase conjugated anti-rabbit (H & L chain specific, Kirkgaard and Perry Laboratories, Gaithersburg, MD) at a dilution of 1∶2000 or anti-mouse antibodies (H & L chain specific, Kirkgaard and Perry Laboratories, Gaithersburg, MD) at a dilution of 1∶2000. Wells were subsequently washed as above and color development was made using the peroxidase substrates, H_2_O_2_ and 2.2′-azinobis (3-ethylbenzthiazolinesulfonic acid)/ABTS (Kirkgaard and Perry Laboratories, Gaithersburg, MD). Optical density (O.D.) was determined at 405 nm and endpoint titers were calculated and graphed using Sigma Plot 10. End point titers were calculated using the serum dilutions that gave an O.D. of 0.2, which is greater than 4 fold of background O.D. absorbance obtained using normal mouse or rabbit serum.

### Expression and purification of recombinant MSP1 C-terminal subunit proteins in *Drosophila* S2 cell

The recombinant MSP1 C-terminal subunit proteins were produced in the *Drosophila* S2 expression system. The expression system consists of the *Drosophila* S2 cells [42] and a series of broad host plasmid vectors that directed the expression of heterologous proteins [43]. The expression plasmid, pMttbns (derived from pMttPA) contains the following elements: *Drosophila melanogaster* metallothionein promoter, the human tissue plasminogen activator secretion leader (tPAL) and the SV40 early polyadenylation signal. A 14 base pair BamHI fragment was excised from the pMttbns vector to yield pMttΔXho creating a unique XhoI. This expression vector results in the secretion of the target protein into the culture medium. The MSP-1 sequences were introduced into the pMttΔXho vector using the unique BglII and XhoI sites.

**Figure 1:**
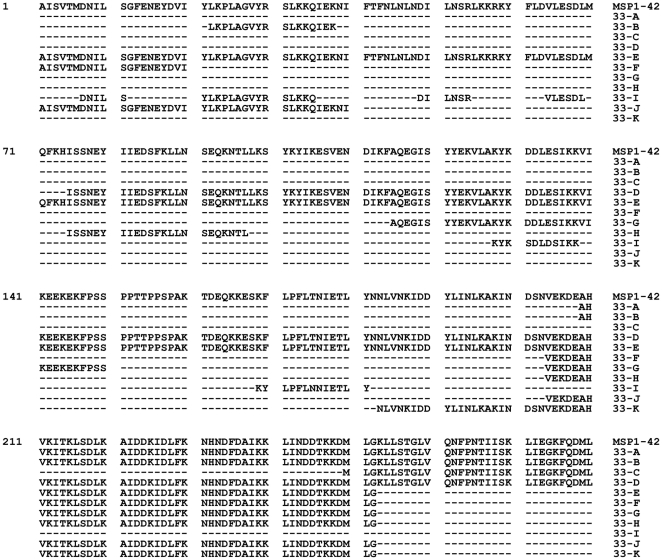
Aligned amino acid sequences of the eleven truncated MSP1-42 subunit protein constructs compared to MSP1-42. All constructs contain the MSP1-19 fragment (not shown) at the C-terminal end.

Eleven constructs, referred hereto as Constructs 33-A – 33-K, were designed to express regions of MSP1-33 fused to MSP1-19 (Figure 1). These constructs were selected based on T-cell epitope predictions via the computer algorithm, Propred [44] or empirically by antigen driven human PBMC proliferation assays [28], [29], [30]. Table 1 shows the amino acid sequence of the identified and predicted T cell epitopes used in the design of the Constructs 33-A – 33-K. For the construction of Constructs 33-A – 33-K expression plasmids, two strategies were used either separately or in combination. The first strategy utilized PCR amplified DNA sequences encoding T cell epitope regions from the MSP1-42 fragment that was derived from the FUP strain genomic DNA (Table 2). The second strategy utilized oligonucleotides encoding for the entire T cell epitope fragment(s) (Table 2). All PCR and oligonucleotide generated MSP1 C-terminal subunit gene fragments were designed to include restriction endonuclease sites (BamHI and XhoI) that were used for cloning.

**Table 1:** Sequence and location of previously identified and predicted T cell epitopes in truncated Constructs 33-A - 33-K.

	MSP1-33 Amino Acid Position N-base #	Amino Acid Sequence
**Identified T cell epitopes**		
1	3-19	SVTMDNILSGFENEYDV
2	22-38	LKPLAGVYRSLKKQIEK
3	37-54	EKNIFTFNLNLNDILNSR
4	81-95	IIEDSFKLLNSEQKN
5	118-134	GISYYEKVLAKYKDDLE
6	127-145	AKYKDDLESIKKVIKEEKE
7	175-191	TNIETLYNNLVNKIDDY
8	190-202	DYLINLKAKINDS
9	210-223	HVKITKLSDLKAID
10	225-244	KIDLFKNHNDFDAIKKLIND
11	252-270	GKLLSTGLVQNFPNTIISK
12	257-275	TGLVQNFPNTIISKLIEGK
13	263-275	FPNTIISKLIEGKFQDML
**Predicted T cell epitopes**		
14	22-30	LKPLAGVYR
15	69, 128-135	LKYKSDLDS
16	170-178	YLPFLNNIE
17	9-11, 21-26	ILSYLKPLA
18	29-35, 49-50	YRSLKKQDI
19	174-181	LNNIETLY
20	32-35, 49-53	LKKQDILNS
21	65-69, 128-131	LESDLKYKS
22	177-181	IETLY
23	50-54, 64-67	ILNSRVLES
24	19-37	VIYLKPLAGVYRSLKKQIE
25	43-56	FNLNLNDILNSRLK
26	69-85	LMQFKHISSNEYIIEDS
27	170-189	FLPFLTNIETLYNNLVNKID
28	180-201	LYNNLVNKIDDYLINLKAKIND

**Table 2:** Primer Sequences for the Construction of Truncated MSP1-42 Constructs.

Construct	MSP1-33 (Amino Acid Position, N → C)	Primers/Oligonucleotides
33-A	209-280	F: acgtacggatccgttggcggtggtaccGCACATGTTAAAATAACTAAAC
		R: agtacaatctcgagttactaACTGCAGAAAATACCATCGAAAAGTG
33-B	22-38	F: aaagttggcggtggtaccGCACATGTTAAAATAACTAAAC
		R: agtacaatctcgagttactaACTGCAGAAAATACCATCGAAAAGTG
	209-280	F: acgtacggatccgttggcggtggtaccGCACATGTTAAAATAACTAAAC
		R: agtacaatctcgagttactaACTGCAGAAAATACCATCGAAAAGTG
33-C	250-280	F: acgtacggatccgttggcggtggtaccATGCTTGGCAAATTACTTAG
		R: agtacaatctcgagttactaACTGCAGAAAATACCATCGAAAAGTG
33-D	76-280	F: tagcggatccACACTTTTAAAAAGTTACAAA
		R: agtacaatctcgagttactaACTGCAGAAAATACCATCGAAAAGTG
33-E	1-252	F: gtcgactagtatgGCAATATCTGTCACAATGGAT
		R: gctacggccatggcggcggcggcggTTCGTATAGAAAAAAGCA
33-F	1-20	F: actagtatgGCAATATCTGTCACAATGGATAATATCCTCTCAGGATTTGAAAATGAATATGATGTTATAggcggcggc
		R:ctaggcggcggcggATATTGTAGTATAAGTAAAAGTTTAGGACTCTCCTATAATAGGTAACACTGTCTATAACGGTAT
	204-252	F: atcgactagtggcggcggcggatccggcGTTGAAAAAGATGAAGCACAT
		R: gctacggccatggcggcggcggcggTTCGTATAGAAAAAAGCA
33-G	115-150	F: gtcgactagtatgGCACAGGAAGGTATAAGTTAT
		R: gctacggcctaggcggcggcggACTACTACCCTTGAAGAGGAA
	204-252	F: atcgactagtggcggcggcggatccggcGTTGAAAAAGATGAAGCACAT
		R: gctacggccatggcggcggcggcggTTCGTATAGAAAAAAGCA
33-H	75-97	F:CTAGTATGATATCCTCAAATGAATACATTATTGAAGATTCATTTAAATTATTGAATTCAGAACAAAAAAACACACTTGGCGGCGGCG
		R:ctaggTTCACACAAAAAAACAAGACTTAAGTTATTAAATTTACTTAGAAGTTATTACATAAGTAAACTCCTATA
	204-252	F: atcgactagtggcggcggcggatccggcGTTGAAAAAGATGAAGCACAT
		R: gctacggccatggcggcggcggcggTTCGTATAGAAAAAAGCA
33-I	7-11	GATAATATCCTCTCA
	21-36	TATTTAAAACCCTTTAGCTGGAGTATATAGAAGCTTAAAAAAACAAATT
	51-55	TTAAATTCACGTCTT
	64-69	GTATTAGAATCTGATTTA
	128-137	AAATATAAGGATGATTTAGAATCAATTAAA
	159-180	GCAAAAACAGACGAACAAAAGAAGGAAAGTAAGTTCCTTCCATTTTTAACAAACATTGAGACCTTA
33-J	1-40	F:actagtatgGCAATATCTGTCACAATGGATAATATCCTCTCAGGATTTGAAAATGAATATGATGTTATA
		R: gctacggcctaggcggcggcggTACAAAAAAAGTTAAACAAAA
	204-252	F: atcgactagtggcggcggcggatccggcGTTGAAAAAGATGAAGCACAT
		R: gctacggccatggcggcggcggcggTTCGTATAGAAAAAAGCA
33-K	183-252	F: actagtatgAACTTAGTTAATAAAATTGACGATTACTTAATT
		R: gctacggccatggcggcggcggcggTTCGTATAGAAAAAAGCA

S2 cells were cultured in Excel 420 serum free medium (SAFC, St. Louis, MO). The cells were co-transformed with the pMttΔXho-MSP1 expression plasmids and the pCoHygro selection plasmid, which encodes hygromycin resistance utilizing the calcium phosphate co-precipitation method (Invitrogen Kit, Carlsbad, CA) according to the manufacturer's recommendations. Cells were co-transformed with 20 µg total DNA with a 20∶1 ratio of expression plasmid to selection plasmid. Transformants were selected with hygromycin B (Roche Molecular Biochemicals, Indianapolis, IN) at 300 µg/mL. For expression studies, cells were induced with 200 µM CuSO_4_. The recombinant proteins were purified from the culture supernatant by immunoaffinity chromatography utilizing the mAb 5.2 [45], and analyzed by SDS-PAGE.

### Immunization with recombinant truncated MSP1-42 subunit proteins

Swiss Webster mice were divided into 11 different vaccination groups (12 mice per group). Each mouse group was immunized with a different truncated MSP1-42 construct and two control groups were immunized with either MSP1-19 or MSP1-42. All mice were immunized three times at 21 days intervals, via the IP route. The first immunization consisted of a sub-optimal dose of 2 µg antigen, followed by two booster injections with an optimal dose of 5 µg [46]. The immunogens were emulsified in Complete Freund's Adjuvant (CFA) for the primary injections and in Incomplete Freund's Adjuvant (IFA) for booster injections. Sera were obtained through tail bleeds, 14 days after each immunization.

New Zealand White rabbits were divided into 10 different immunization groups (3 rabbits per group). NZW rabbits were immunized with S2 cell expressed recombinant truncated MSP1-42 proteins formulated in Montanide ISA51 adjuvant. Each dose of vaccine composed of 50 µg of antigen in 250 µl PBS, and was emulsified with an equal volume of ISA51 as per the manufacturer's recommendations. The emulsion was injected, via the IM route, into the left and right thighs. A total of four immunizations were given at 4 weeks intervals and sera collected 21 days after the last immunization were used in ELISAs and parasite growth inhibition assays. As control, rabbits were similarly immunized with S2 cell expressed full length MSP1-42.

### ELISPOT Assay

ELISPOTS of splenocytes from immunized mice were performed according to methods previously described [47]. Ninety-six well PVDF plates (Millipore Inc., Bedford, MA) were coated with 10 ug/mL of anti-IFN-γ mAb (R4-642) and 5 ug/mL of anti-IL-4 mAb (11B11) (BD Biosciences, San Diego, CA) and incubated overnight at room temperature. Plates were then washed five times with sterile phosphate buffered saline (PBS) and blocked for 60 minutes at 37°C with DMEM/10% fetal bovine serum. Mice from each vaccination group were sacrificed by cervical dislocation, the spleen removed, and placed in DMEM. The spleen was crushed and individual suspensions of splenocytes were prepared by passing through a cell strainer and washing four times in DMEM. Splenocytes were plated at 0.5×10^6^ cells/well, 0.25×10^6^ cells/well, and 0.125×10^6^ cells/well, and the corresponding recombinant immunogen was added at a final concentration of 20 ug/mL as the stimulating antigen. Positive control wells were incubated with 5 ng/mL of phorbol myristate acetate (PMA) and 1 ng/mL ionomycin. Plates were incubated again at 37°C for 48 hours and then processed by washing four times with PBS and five times with PBS with 0.05% Tween-20. Biotinylated monoclonal antibodies against IFN-γ at 2 µg/mL (XMG1.2), and monoclonal antibodies against IL-4 at 1 µg/mL (BVD6-24G2) (BD, Biosciences, San Diego, CA) were added to appropriate wells and incubated for three hours at 37°C. Plates were again washed as mentioned above and incubated with peroxidase conjugated streptavidin (Kirkgaard and Perry Laboratories, Gaithersburg, MD) for 60 minutes at a concentration of 1∶800. After seven washings, plates were developed with a solution consisting of 3,3′-Diaminobenzidine tetrahydrochloride (DAB) (Sigma-Aldrich St. Louis, MO, 1 mg/ml) and 30% H_2_O_2_ (Sigma-Aldrich St. Louis, MO). Cytokine producing cells were counted microscopically and data presented as spot-forming-units (SFU) per million of plated splenocytes.

### *In vitro* parasite growth inhibition assays

The ability of rabbit sera generated by immunization with each of the nine truncated MSP1-42 subunit proteins to inhibit parasite growth was determined using an *in vitro* assay [20], [32], [48], [49]. The inhibition assay was performed using sorbitol synchronized parasite cultures (3D7 strain) as described [32]. Synchronized parasite cultures at a starting parasitemia of 0.2% and 0.8% hematocrit were incubated in 30% heat inactivated immune sera. Cultures were then incubated for 72 hours with periodic mixing. Parasitemia was then determined microscopically by Giemsa staining. The degree of parasite growth inhibition was determined by comparing the parasitemias of cultures incubated with pre-immune sera as previously described [32], [48], [49].

### *In vitro* assay for blocking antibodies

To test for the presence of blocking antibodies that interfere with anti-MSP1 growth inhibitory antibodies, synchronized parasite cultures were incubated in a mixture of 20% heat inactivated anti-Construct 33-C sera and 15% anti-MSP1-42 inhibitory sera as previously described [50]. Normal rabbit sera were similarly mixed with the inhibitory anti-MSP1-42 sera as control. The inhibitory anti-MSP1-42 sera was obtained from a previous vaccination study [39]. In that study we produced highly inhibitory anti-MSP1-42 antibodies (>90% growth inhibition) by hyper-immunization of rabbits with full length MSP1-42 emulsified in CFA [39]. These sera were used because of their very high levels of parasite growth inhibition making their inhibitory activities less prone to dilution effects when mixing with other sources of rabbit sera.

### Data handling and Statistics

Sigma Plot 10® and GraphPadPrism 4® were used to calculate end point titers. One-way Analysis of Variance (ANOVA) and Student t-test were used to determine significant differences in antibody titers amongst the different test groups. Cytokine responses (ELISPOT) in mice and parasite growth inhibition by different anti-truncated MSP1-42 sera were analyzed by Logistic Regression for Repeated Measures and Fisher Exact Test: respectively (IBM SPSS Statistics). A p<0.05 was considered statistically significant.

## Results

### Expression of recombinant proteins

**Figure 2:**
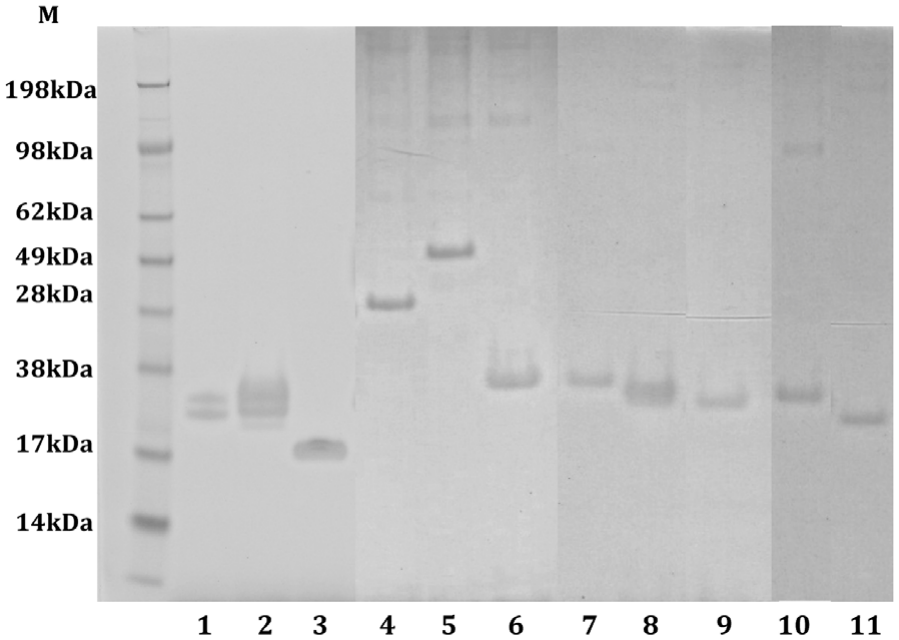
SDS-PAGE of the purified S2 cell expressed truncated MSP1-42 proteins. Expected molecular sizes of each construct are in parenthesis. Lane 1: Construct 33-A (19 kDa); Lane 2: Construct 33-B (21 kDa); Lane 3: Construct 33-C (14 kDa); Lane 4: Construct 33-D (29 kDa); Lane 5: Construct 33-E (32 kDa); Lane 6: Construct 33-F (39 kDa); Lane 7: Construct 33-G (21 kDa); Lane 8: Construct 33-H (19 kDa); Lane 9: Construct 33-I (17 kDa); Lane 10: Construct 33-J (21 kDa); Lane 11: Construct 33-K (19 kDa).

**Figure 3:**
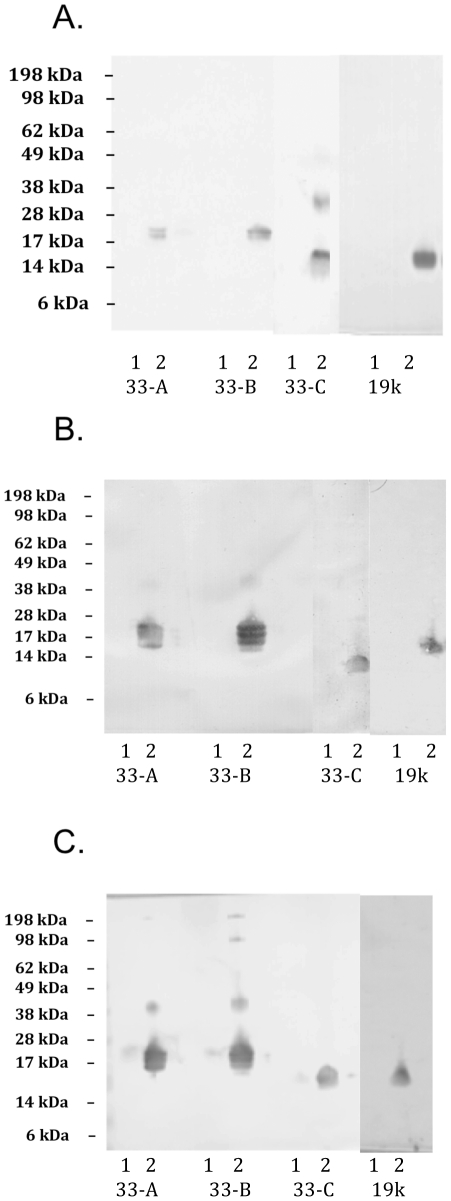
Truncated MSP1-42 proteins possess disulfide sensitive conformation. Immunoblots of recombinant proteins separated under reducing (lanes 1) and non-reducing (lanes 2) conditions and probed with conformational sensitive anti-MSP1-19 monoclonal antibodies. Panel A: Construct 33-A – 33-C and MSP1-19 probed with mAb 12.8; Panel B: Constructs 33-A – 33-C and MSP1-19 probed with mAb 2.2; and Panel C: Constructs 33-A – 33-C and MSP1-19 probed with mAb 5.2 [47], [48].

Induced culture supernatants from *Drosophila* S2 cells transformed with Construct 33-A – 33-K were clarified and the recombinant proteins were purified by immuno-affinity chromatography. The purified proteins were analyzed by SDS-PAGE. A representative reducing gel of the purified proteins is shown in Figure 2. A protein doublet was observed after purification of Construct 33-A (∼19 kDa) and 33-B (∼21 kDa) (Figure 2, Lanes 1 and 2), which may result from different degrees of glycosylation in the insect cells. A single protein band was observed after purification of Construct 33-C – 33-K, with molecular sizes of ∼14, 29, 32, 39, 21, 19, 17, 21, and 19 kDa; respectively (Figure 2, Lanes 3-11). All truncated MSP1 C-terminal recombinant subunit proteins maintained native-like conformation based on binding with conformationally sensitive monoclonal antibodies. As examples, Construct 33-A, 33-B, and 33-C were reactive to MSP1-19 specific monoclonal antibody, mAb 5.2 [51], mAb 12.8, and mAb 2.2 [52] on immunoblots when prepared under non-reducing conditions. These same antibodies did not bind to the recombinant subunit proteins when prepared under reducing conditions (Figure 3). By ELISAs, conformational dependent mAb 5.2 reacted equally well with all eleven constructs as with MSP1-42 (data not shown). This suggests that all constructs retained similar conformation as MSP1-42.

### Immunogenicity of the truncated MSP1-42 subunit proteins in mice

**Figure 4:**
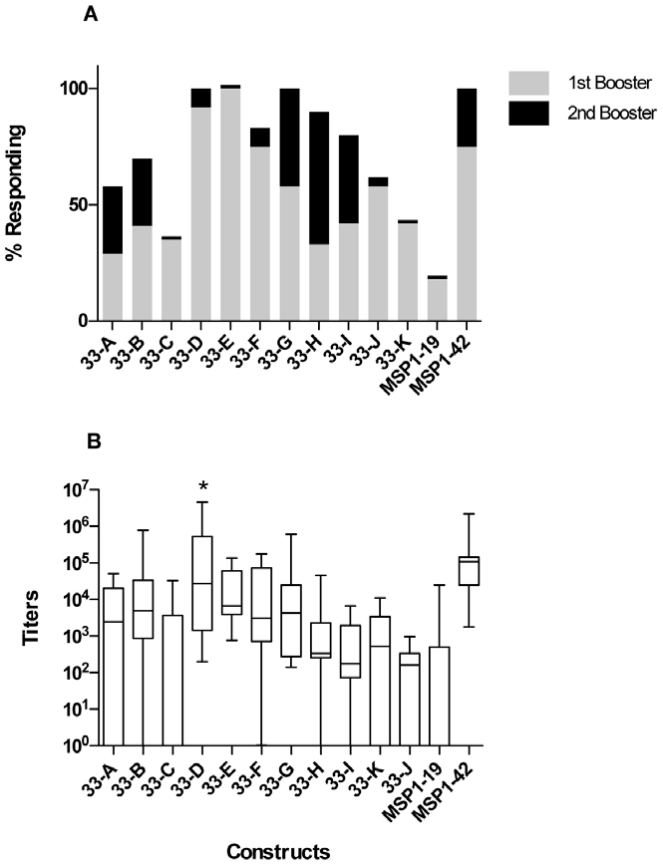
ELISA antibody responses against MSP1-19 in Swiss Webster mice immunized with recombinant truncated MSP1-42 proteins. Panel A, percent responsiveness of mice immunized with Constructs 33-A – 33-K after the first booster injection (grey) and after the second booster injection (black). Panel B, antibody titers of mice vaccinated with Constructs 33-A – 33-K. Results of tertiary bleeds are shown. Horizontal bars indicate mean antibody titers. ANOVA (p<0.05) indicated that the levels of antibody titers differed among groups. Asterisk indicates a significant difference (Turkey post-hoc test, p<0.05) between Construct 33-D and all other vaccination groups.

Secondary and tertiary sera from immunized Swiss Webster mice were tested for antibodies specific for MSP1-19 by ELISA. Responders were defined as having an ELISA O.D. of >0.2 at a 1/50 serum dilution. This value is greater than four-fold the O.D. values observed for pre-immune mouse sera. As shown in Figure 4A, the responsiveness to the immunogens varied from a low 30–35% to a high percent response of 92%–100% after two immunizations. In comparison, MSP1-19 had the lowest response rate (18%) of all the constructs. Analysis of the tertiary sera, however, revealed that an additional immunization (Figure 4A, black bars) was able to increase the number of responders for the majority of the constructs with the exception of Construct 33-C, Construct 33-K, and MSP1-19. Construct 33-D – 33-I induced response rates similar or comparable to the full length MSP1-42 (Figure 4A).

The immunogenicity of the truncated MSP1-42 proteins was also evaluated in terms of MSP1-19 specific antibody titers. In each of the antigen groups there were high and low responders (Figure 4B). High responders were defined as having an ELISA O.D. of >0.6 at a 1/1250 serum dilution. There were significant differences in antibody titers across the vaccination groups (One-way ANOVA [F(10, 133) = 2.345, p = 0.014]). Construct 33-D induced significantly higher antibody titers than all other truncated constructs (Tukey post-hoc comparison, p<0.05).

### Regions of MSP1-33 influenced cytokine responses

**Figure 5:**
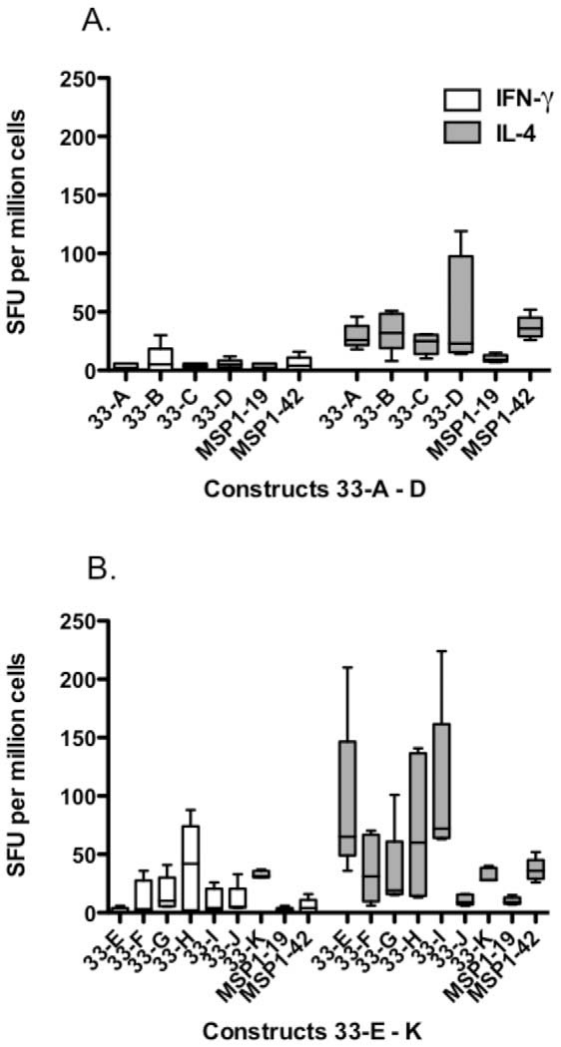
Induction of MSP1-specific IL-4 (grey bars) and IFN-γ (white bars) responses, as determined by ELISPOT, in mice immunized with truncated MSP1-42 proteins. Panel A: Constructs 33-A – 33-D; Panel B: Constructs 33-E – 33-K. Horizontal bars indicate mean SFU. Logistic Regression for Repeated Measures indicated that IFNγ levels were significantly higher (p<0.05) in Construct 33-E – 33-K compared to Construct 33-A – 33-D. No significant difference was found when comparing IL-4 levels. Mouse splenocytes were harvested 21 days after the last immunization.

Splenocytes of immunized mice were stimulated *in vitro* with the immunogens and analyzed by IL-4/IFN-γ ELISPOTS (Figure 5A and B). For the purpose of analysis, constructs were separated into two groups, Construct 33-A – 33-D and Constructs 33-E – 33-K, basing on the fact that the Construct group 33-E – 33-K does not contain T cell epitopes within the 31 amino acid sequence immediately N-terminal of the MSP1-19. Accordingly, Construct group 33-E – 33-K induced significantly higher levels of IFNγ than Construct group 33-A – 33-D (Logistic Regression for Repeated Measures, p<0.05). No significant difference between the two groups was observed for the production of IL-4. Thus, Constructs containing T cell epitopes within the 31 amino acid sequence induced a skewed TH2 response (Figure 5A); whereas, those without this sequence induced a more balanced TH1/TH2 response (Figure 5B). Constructs 33-J and 33-K were not further studied since mouse data indicated that they did not induce high responsiveness or immunogenicity.

### Immunogenicity of the truncated MSP1-42 subunit proteins in rabbits

Rabbit sera from quaternary bleeds were tested by ELISA for MSP1-19 and MSP1-42 specific antibodies. All nine constructs were able to induce an antibody response (Table 3). When antibody endpoint titers were analyzed among the nine truncated MSP1-42 constructs, Construct 33-C induced the highest mean antibody titers (geometric mean) against both MSP1-19 and MSP1-42; whereas, Construct 33-F induced the lowest mean antibody titers. Construct 33-D and 33-I had significantly lower mean antibody titers than MSP1-42 (p = 0.02 and p = 0.0003; respectively). In addition, Construct 33-I also had significantly lower titers than Construct 33-C (p = 0.02).

**Table 3:** In vitro Parasite Growth Inhibition of Rabbit Anti-truncated MSP1-42 Sera.

Rabbit Sera (4^th^ Bleeds)		% Parasite Growth Inhibition^a^	Reciprocal ELISA Antibody Titers			
			MSP1-42	Mean (Rbt#1-3±SD)^ b^	MSP1-19	Mean (Rbt#1-3±SD)^ b^
Anti-33-A^d^	Rbt #1	75%	624,000	482,000±131,538	93,000	43,000±37,233
	Rbt #2	37%	361,000		23,000	
	Rbt #3	24%	498,000		36,000	
Anti-33-B	Rbt #1	66%	27,000	101,000±109,610	161,000	139,000±20,133
	Rbt #2	26%	156,000		137,000	
	Rbt #3	0%	245,000		121,000	
Anti-33-C	Rbt #1	10%	2,490,000	1,440,000±814,338	254,000	365,000±122,111
	Rbt #2	32%	948,000		385,000	
	Rbt #3	22%	1,265,000		498,000	
Anti-33-D^c^	Rbt #1	58%	93,000	218,000±450,617	43,000	146,000±269,367
	Rbt #2	58%	125,000		133,000	
	Rbt #3	94%	889,000		548,000	
Anti-33-E	Rbt #1	31%	79,000	340,000±2,957,000	27,000	111,000±1,026,000
	Rbt #2	71%	113,000		28,000	
	Rbt #3	53%	5,218,000		1,804,000	
Anti-33-F^d^	Rbt #1	56%	137,000	55,000±59,355	117,000	74,000±41,053
	Rbt #2	0%	54,000		93,000	
	Rbt #3	0%	22,000		37,000	
Anti-33-G^d^	Rbt #1	0%	156,000	180,000±58,774	202,000	220,000±70,887
	Rbt #2	0%	253,000		172,000	
	Rbt #3	0%	22,000		307,000	
Anti-33-H^d^	Rbt #1	26%	110,000	169,000±61,101	223,000	214,000±21,362
	Rbt #2	56%	190,000		190,000	
	Rbt #3	0%	230,000		230,000	
Anti-33-I^c^ ^d^	Rbt #1	85%	125,000	133,000±11,150	109,000	109,000±11,015
	Rbt #2	78%	146,000		121,000	
	Rbt #3	66%	129,000		99,000	
Anti-MSP1-42	Rbt #1	60%	1,252,000	1,198,000±162,263	140,000	179,000±105,510
	Rbt #2	0%	1,024,000		317,000	
	Rbt #3	56%	1,338,000		129,000	

a Means of two growth inhibition assays.

b Geometric mean and standard deviation of antibofy titers.

c Fisher Exact Test, p<0.05.

d Consider titer significantly lower then MSP1-42 ((p = 0.0021), 33-F(p = 0.0195), 33-G(p = 0.0004), 33-H(p = 0.0003), 33-I(p = 0.0003).

### *In Vitro* parasite growth inhibitory activity of truncated anti-MSP1-42 antibodies

The ability of the rabbit sera generated by immunizations with the truncated MSP1-42 constructs, formulated with ISA51, to inhibit *in vitro* parasite growth was evaluated using an *in vitro* assay [20], [32], [48], [49]. Inhibition greater than 50% is considered to be biologically significant [19], [53], [54]. As shown in Table 3, there were constructs which induced no significant inhibitory antibodies in the immunized rabbits (Construct 33-C and 33-G). There were constructs with which one or two of the three immunized animals produced significant levels of inhibitory antibodies (Construct 33-A, 33-B, 33-D, 33-E, 33-F, and 33-H). The positive control, full length MSP1-42, had two out of three animals inducing significant levels of inhibitory antibodies. Construct 33-D and 33-I were the only two immunogens able to induce significant levels of inhibitory antibody in three out of three animals (Table 3). The ability of Construct 33-D and 33-I to induce inhibitory antibodies greater than 50% in rabbits were found to be significant as compared to other construct groups (Fisher Exact Test, two sided p-value  = 0.0051) (Table 3). It is also important to emphasize that Construct 33-C failed to induce significant inhibitory antibodies, despite producing the highest mean antibody titers. On the other hand, anti-Construct 33-I antibodies had the highest mean percent parasite inhibition (76%) despite having ELISA titers that were at least one log lower than those produced by Construct 33-C and the full length MSP1-42 (p = 0.02). To a lesser extent, Construct 33-D also induced significant parasite inhibition despite that fact that antibody titers were significantly lower than MSP1-42 (p = 0.02).

### Anti-Construct 33-C antibodies interferes with inhibitory anti-MSP1-42 antibodies

Non-inhibitory anti-Construct 33-C antibodies were tested for interfering/blocking effects on inhibitory MSP1-42 sera. The highly inhibitory MSP1-42 sera were obtained from previous vaccination studies in which rabbits were hyper-immunized with full length MSP1-42 emulsified in CFA [39]. The data in Table 4 demonstrates that when anti-MSP1-42 sera with high levels of inhibitory activity, were mixed with anti-Construct 33-C sera from two different rabbits (anti-Construct 33-C sera #1 and anti-Construct 33-C sera #2) the levels of parasite growth inhibition were reduced. MSP1-42 inhibitory serum #1 alone had an 86% inhibition of parasite growth. The addition of anti-Construct 33-C serum #1 to MSP1-42 inhibitory serum #1 decreased the parasite growth inhibition from 86% to 59%. The addition of anti-Construct 33-C serum #2 reduced growth inhibition from 86% to 73%. Similarly for MSP1-42 inhibitory serum #2, which alone inhibited parasite growth at 93%, the level of p parasite inhibition was reduced from 93% to 73% when anti-Construct 33-C serum #1 was added; and from 93% to 89% when anti-Construct 33-C serum #2 was added. The data also shows that anti-Construct 33-C serum #1 had higher blocking/interfering activity than anti-Construct 33-C serum #2. Mixing of normal rabbit serum with the MSP1-42 inhibitory sera had negligible effects on parasite inhibition.

**Table 4:** Anti-Construct 33-C Antibodies Interfere with Inhibitory Anti-MSP1-42 Antibodies.

Serum Samples	% Parasite Growth Inhibition
**MSP1-42 Inhibitory Serum #1**	
alone	86%
+Normal Rabbit Serum	85%
+Anti-Construct 33-C Serum#1	59%
+Anti-Construct 33-C Serum#2	73%
**MSP1-42 Inhibitory Serum #2**	
alone	93%
+ Normal Rabbit Serum	91%
+Anti-Construct 33-C Serum#1	73%
+Anti-Construct 33-C Serum#2	89%

## Discussion

The development of recombinant MSP1-based malaria vaccines to date has primarily focused on MSP1-42 and its C-terminal sub-fragment, MSP1-19. The main purpose of this study is to examine immune responses to the N-terminal sub-fragment of MSP1-42, MSP1-33, in order to better understand its relevance and potential in enhancing the immunogenicity and efficacy of MSP1-42 based vaccines.

Previous studies have shown that MSP1-19 has limited ability in inducing an antibody response in a genetically diverse host population [31], [33]. This is thought to be due to the scarcity of T helper epitopes on MSP1-19 [29], [31]. Although inclusion of additional heterologous T cell epitopes may overcome this limitation [55], such vaccines lack the advantage of priming cognate T cell help that can be recalled during natural infections. A number of T cell epitopes have already been identified on MSP1-33 [28], [29], [30], [56], and many of these epitopes were included in the eleven recombinant subunit constructs described here. Previous studies of these T cell epitopes have only focused on T cell proliferation and/or cytokine production [28], [29], [30]. Whether these epitopes can provide functional “help” to enhance anti-MSP1-19 antibody responses have not been investigated. Our results demonstrated that all truncated MSP1-33 fragments, when fused to MSP1-19, were able to broaden the antibody responsiveness to MSP1-19 in outbred mice as compared to MSP1-19 alone. But the degree in broadening responsiveness varied among the different fragments. A number of constructs were able to induce a generalized response (80% -100% response rate), which were comparable/equal to MSP1-42 (Figure 4A). This suggests that some of the T cell epitope regions on MSP1-33 of *P. falciparum* can provide adequate levels of helper function for the induction of antibodies in a genetically diverse population. A previous study with *P. yoelii* shows that MSP1-33 can provide help in the induction of anti-MSP1-19 antibodies [57]. However, this study only focused on Balb/c restricted haplotype and did not address the ability to broaden the response in a population of diverse MHC makeup. Our data provides for the first time, experimental validation of the long-held assumption that MSP1-33 possesses T helper epitopes that can enhance antibody responses specific for MSP1-19 in a genetically diverse population.

Aside from providing T helper functions, our studies indicate that the T cell epitope regions of MSP1-33 critically affected the quality of the anti-MSP1-19 responses. This linkage of T cell help to B cell specificity has been previously observed in a number of studies [35], [36], [37], [38] and more recently it has been extended to large protein molecules [58]. One measurement of the specificity of the anti-MSP1 antibody responses is their ability to inhibit parasite growth in vitro. Accordingly, inclusion of certain T cell epitope regions may contribute positively or negatively towards the induction of inhibitory antibodies. As examples, Construct 33-D and 33-I consistently induced high levels of parasite inhibitory antibodies; whereas, Construct 33-C failed to induce appreciable amount of inhibitory antibodies despite producing high antibody titers. This suggests that the T cell epitope regions in Construct 33-C were unable to focus antibody responses to inhibitory epitopes. These inhibitory antibody responses observed are due to anti-MSP1-19 antibodies since negligible anti-MSP1-33 antibodies were induced (data not shown). Furthermore, the antibodies induced by Construct 33-C interfered with the parasite inhibitory activity of anti-MSP1-42 antibodies (Table 4). The MSP1-33 specific T cell epitope regions also influenced the relative balance of TH1 versus TH2 responses. Inclusion of the 31 amino acid sequence from Construct 33-C in other truncated MSP1-42 constructs had a tendency to bias response towards the TH2 arm (Figure 5). Thus, based on our antibody and ELISPOT analyses the T cell epitope regions contained in Construct 33-C would not be beneficial because of their tendency to potentiate undesirable antibody and T cell responses. The negative effects of these T cell epitopes may be modulated by virtue of their relative dominance when presented with other MSP1-33 specific T cell epitopes in outbred populations, and this could be the situation observed with other constructs in our study. Since it is difficult to predict and/or anticipate the relative dominance of T cell epitopes in a genetically diverse population, it may be prudent to preemptively eliminate the 33-C specific T cell epitopes from vaccine design in order to avoid production of undesirable antibodies and T cell responses. Accordingly, constructs such as 33-A, 33-B, and 33-D may possibly be made more effective as an immunogen by eliminating the T cell epitope regions found in Construct 33-C. Along the same line, since the T cell epitopes within Construct 33-C and Construct 33-I are recognized by humans from malaria endemic populations [28], [29], [30], selective exclusion and/or inclusion of these epitopes from a MSP1-42 based vaccine would ensure boosting of only the desirable preexisting anti-MSP1 responses, which in turn may enhance overall vaccine efficacy. Epidemiological studies have provided evidence that protective immunity afforded by MSP1 is dependent on the production of inhibitory antibodies [5], [59], [60], [61], [62]. However, other studies have argued the lack of correlation between anti-MSP1 inhibitory antibodies alone with malaria immunity [28], [63], [64] and protective anti-MSP1-19 response may involve other immune effector mechanisms such as Antibody Dependent Cell Cytotoxicity (ADCC), which involves Fc-dependent killing of parasites through neutrophils and macrophages [65], [66]. It is important to point out that although the present study demonstrated the influence of MSP1-33 specific T cell epitopes on the induction of parasite inhibitory antibodies, it is possible that these T cell epitopes may have a broader influence on the development of other protective anti-MSP1-42 immune effector responses.

Recently, a prime-boost immunization regimen utilizing simian adenoviral and poxviral vectors expressing four N-terminal conserved blocks of MSP1 fused with both dimorphic forms of MSP1-42 was reported as a new candidate malaria vaccine [67]. These vaccines were found to induce high antibody titers against MSP1 and have high growth inhibitory activities [67]. The study did not examine the contribution of MSP1-42 specific T cell epitopes to the development of inhibitory antibodies. Further, since the N-terminal regions (Blocks 1, 3, 5, 12) are physically separated from MSP1-42 during merozoite development and invasion it may be difficult for these regions to provide cognate help in inducing or boosting antibody responses to MSP1-19. Previous studies have also utilized non-MSP1 derived T cell epitopes in conjunction with the MSP1-19 immunogen to overcome genetic restrictions of MSP1-19 induced protection [55]. The addition of these non-MSP1 T cell epitopes shows an impact on antibody subclass and protective efficacy [55]. However, these epitopes will not be able to boost anti-MSP1-19 antibodies during natural infections. Our strategy of selective inclusion of MSP1-33 T cell epitopes has the advantage of boosting existing immunity to MSP1-42/MSP1-19 via cognate T cell help in malaria exposed populations.

Our results provide a fresh glimpse on the manner by which anti-MSP1-19 antibody response may be modulated during natural infections where a full complement of MSP1-42 specific T cell epitopes is presented. As example, dominant recognition of T cell epitopes within Construct 33-C in malaria-exposed individuals may skew responses toward the development of non-inhibitory and/or interfering types of antibodies. It is tempting to speculate that deployment of a full length MSP1-42 vaccine under this setting, may not be able to potentiate the level(s) of protective immunity and specificity observed with immunizations in naive animal models [17], [18], [19], [20]. Moreover, our results may also help explain the lack of efficacy in a recent MSP1-42 clinical trial in malaria endemic areas.

**Figure 6:**
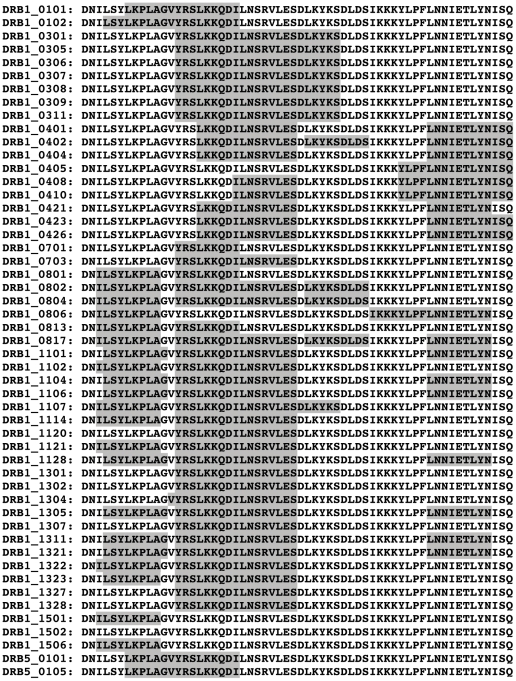
Class II epitope prediction of the sequence of Construct 33-I by computer algorithm (Propred). Grey shaded sequences represent motifs that may bind to Class II molecules.

An important outcome of our study is the identification of a more efficacious MSP1 vaccine than the current full length MSP1-42; namely, Construct 33-I. Construct 33-I, along with Construct 33-D, were the only two immunogens able to induce significant parasite growth inhibitory antibodies (>50%) in all immunized rabbits; whereas all other vaccine groups including MSP1-42 failed to do so. Importantly for Construct 33-I, the levels of parasite inhibition were achieved at much lower antibody titers than what were induced by MSP1-42. The prevailing view of an efficacious MSP1-42 vaccine is the requirement of high antibody titers needed for in vivo protection or in vitro parasite inhibition. This would necessitate the use of powerful adjuvants to achieve the desired immunogenicity. Our data with Construct 33-I indicates that this truncated MSP1-42 vaccine can induce potent anti-parasite antibodies at a much lower overall antibody response. This would eliminate the prerequisite for strong adjuvants for its deployment as a human malaria vaccine. An equally attractive attribute of Construct 33-I is the sequence compositions of T cell epitopes. First, the MSP1-33 specific T cell epitopes in this construct are based entirely of conserved sequences, thereby circumventing the potential complications of allelic variations. Second, computer algorithm analyses of the T cell epitope sequences revealed a promiscuous binding to all major HLA Class II molecules (Figure 6), suggesting a potential broad immune responsiveness that this vaccine can elicit in humans. The superior immunological characteristics that Construct 33-I has over MSP1-42 strongly justify further evaluations as a second generation MSP1-42 based human malaria vaccine. To further validate the vaccine candidacy of Construct 33-D and 33-I, it would be necessary to perform immunogenicity and efficacy studies in non-human primate models. Equally important is to evaluate whether the T cell epitope regions defined by Construct 33-D and 33-I are immunogenic in malaria exposed human populations; and whether human T cells specific for these epitopes will be able to provide necessary helper functions for the induction of protective antibodies.
